# Quantitative and qualitative adenosine perfusion magnetic resonance imaging for the detection of coronary artery disease at 3 Tesla

**DOI:** 10.1186/1532-429X-16-S1-P180

**Published:** 2014-01-16

**Authors:** Dominik Buckert, Volker Rasche, Wolfgang Rottbauer, Peter Bernhardt

**Affiliations:** 1 University of Ulm, Ulm, Germany

## Background

Adenosine perfusion cardiac magnetic resonance imaging (CMR) at 3 Tesla has been suggested to yield superior diagnostic accuracy in comparison to 1.5 Tesla. We sought to evaluate its diagnostic accuracy in a patient cohort with intermediate risk and typical angina symptoms in comparison to quantitative coronary analysis (QCA) and fractional flow reserve (FFR).

## Methods

Eighty-six patients with intermediate risk, angina pectoris, and suspected coronary artery disease were enrolled into the study. All patients were examined in a 3 T CMR system including functional imaging of the left ventricle, adenosine and rest perfusion imaging using a bolus of 0.075 mmol/kg gadolinium based contrast agent (Dotarem, Guerbet, France), respectively. CMR studies were performed within 1 week prior to invasive coronary angiography which included QCA and FFR. Myocardial perfusion reserve (MPR) was calculated and correlated to FFR. Visual analysis of the perfusion sequences were compared to QCA. All measurements were performed by two blinded and experienced observers in consensus.

## Results

Visual CMR analysis resulted in a specificity of 0.88 and sensitivity of 0.96 for the detection of a coronary artery stenosis ≥70% as quantified by QCA on a per patient basis. ROC analysis for MPR yielded specificity of 1.0 and sensitivity of 0.9 for the detection of a coronary artery with reduced FFR (≤0.8) on a per vessel basis.

## Conclusions

Qualitative and quantitative analysis of adenosine perfusion CMR at 3 Tesla are able to detect significant coronary artery disease with high diagnostic accuracies, respectively. 3 Tesla CMR might thus serve as an important non-invasive diagnostic tool in clinical work-up of the evaluation of coronary artery disease.

## Funding

Partly funded by a research grant from Guerbet.

